# Multimodal Fluorescent Polymer Sensor for Highly Sensitive Detection of Nitroaromatics

**DOI:** 10.1038/s41598-019-43836-w

**Published:** 2019-05-13

**Authors:** Vishal Kumar, Binoy Maiti, Mrinmoy Kumar Chini, Priyadarsi De, Soumitra Satapathi

**Affiliations:** 1 Department of Physics, Indian Institute of Technology Roorkee, Roorkee, Uttarakhand 247667 India; 2 Polymer Research Center, Department of Chemical Science, Indian Institute of Science Education and Research, Kolkata, Mohanpur, 741246 India

**Keywords:** Electronic properties and materials, Techniques and instrumentation

## Abstract

Detection of nitroaromatic explosives with high sensitivity and selectivity is extremely important for civilian and military safety. Here, we report the synthesis and multimodal sensing applications of an emissive alanine based dansyl tagged copolymer P(MMA-co-Dansyl-Ala-HEMA) (**DCP**), synthesized by RAFT copolymerization. The fluorescent co-polymer exhibited high sensitivity and selectivity towards conventional nitroaromatic explosives such as DNT, TNT and TNP in solution at lower range of µM level and also with saturated vapor of NACs. The quantum yield of the co-polymer was measured to be very high (Φ_f_ = 77%) which make it an ideal candidate for sensing in solution as well as in vapor phase. The fluorescence signal from **DCP** copolymer gets significantly quenched upon addition of aliquots of DNT, TNT, and TNP. The Stern-Volmer constant was calculated to be very high. The quenching mechanism was further established by fluorescence up-conversion, time-resolved fluorescence and steady state absorption spectroscopy. The energetics of sensing process was calculated by Density Functional Theory (DFT) studies. We also fabricate a thin film polymer sensor which was able to detect nitroaromatic vapors with high selectivity. This opens up the possibility of building a low-cost and light-weight nitroaromatic explosives sensor for field use.

## Introduction

Nitroaromatic explosives as chemical warefares pose serious threat to civilian and military safety, and are also recognized as a toxic contaminants for environmental pollution^1^. These explosives materials are usually nitro substituted compounds such as 2,4,6-trinitrophenol (TNP), 2,4-dinitrotoluene (DNT) and 2,4,6-trinitrotoluene (TNT) and are consist of an intimate mixture of chemical oxidant and reluctant which undergoes highly exothermic decomposition causing serious environmental pollution^2^. The large scale use of these explosives in the recent past has prompted the scientific community to develop novel sensing materials for sensitive and selective detection, both in solution and in vapor phase for national security and environmental concern^3^. In addition, explosive materials especially TNP has been excessively used in several other areas such as burn ointment, dyes, glass and the leather industry for commercial production. The excessive utilization of these hazardous chemicals resulted in environmental accumulation, and is eventually contaminating the soil and aquatic systems. Therefore, there is an urgent need to develop a sensor probe for detecting TNP along with other nitro aromatic compounds.

The conventional explosives detection methods mainly rely on canines or sophisticated instruments such as gas chromatography (GC)^4^, ion mobility spectrometry (IMS)^5^, Raman or surface enhanced Raman scattering^6,7^, high performance liquid chromatography (HPLC)^8^, surface Plasmon resonance^9^, and cyclic voltammetry^10^. However, all these methods are expensive, complex and may not be readily translated for field use^11^. On the other hand, fluorescence quenching based optical sensing have attracted great attention owing to its’ high sensitivity, simplicity, and viability both in solution as well as in vapor phases^12^.

In the recent years, numerous fluorescent probes including small molecule, nanoparticles, nano-fibers, Molecular Imprinted Polymer (MIP), Polymeric composites, Metal-Organic Framework (MOF) and fluorescent co-polymers have been developed for the rapid detection of trace amounts of nitroaromatic (NACs) by fluorescence quenching methods^13–19^. Among these, fluorescent polymers are particularly interesting for fluorescence based rapid detection as they exhibit large signal amplification due to the delocalization and rapid diffusion of excitons throughout the individual polymer chains, the so-called molecular wire effect, or one point contact and multipoint response effect, in solution and in thin films^20–22^. Moreover, the fluorescence polymers having comparable energy levels (HOMO & LUMO) with analytes can act as an efficient electron donor^23–28^. On the other hand, NACs have electron-withdrawing nitro groups on the aromatic ring which reduce the energy of the empty π* orbitals rendering them as good electron acceptors^29,30^. The enhanced interaction due to favorable redox potential and rapid electron transfer between electron-rich fluorescent co-polymers to NACs render these fluorescent co-polymers as efficient optical sensor.

Here, we report the synthesis and multimodal sensing applications of a good emissive alanine based dansyl tagged copolymer P(MMA-*co*-Dansyl-Ala-HEMA) (**DCP**), synthesized by RAFT copolymerization. The fluorescent co-polymer exhibited high sensitivity and selectivity towards conventional nitroaromatic explosives such as DNT, TNT and TNP in solution at lower range of μM level and also with saturated vapour of NACs. The quantum yield of the co-polymer was measured to be very high (Φ_f_ = 77%) which make it an ideal candidate for sensing in solution as well as in vapor phase. In solution, upon addition of trace amount of DNT, TNT and TNP fluorescence quenched noticeably by photo-induced electron-transfer. The sensitivity of fluorescence quenching is quantified by plotting Stern–Volmer plot. The quenching mechanism was further confirmed by fluorescence up-conversion, time-resolved fluorescence and steady state absorption spectroscopy. The quenching was found to be combination of static and dynamic in nature. To explore the possibility of using the fluorescent co-polymer as sensor array, drop-casted thin film of **DCP** was fabricated. The fluorescence intensity reduced significantly in real time with exposure to nitroaromatics vapor. The selectivity of **DCP** towards NACs has been tested with various analogues analytes.

## Results and Discussion

### Structural and optical characterization

**Figure 1:**
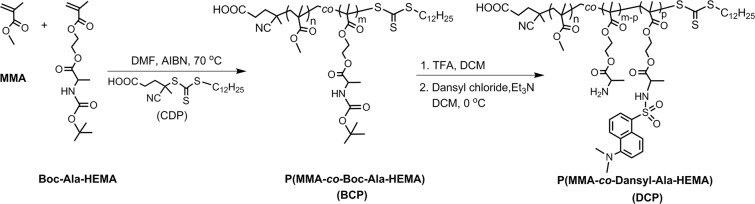
Synthesis of P(MMA-*co*-Boc-Ala-HEMA) (BCP) copolymer followed by deprotection of Boc groups, and subsequent dansyl tagging (P(MMA-*co*-Dansyl-Ala-HEMA) (**DCP**)).

**Figure 2:**
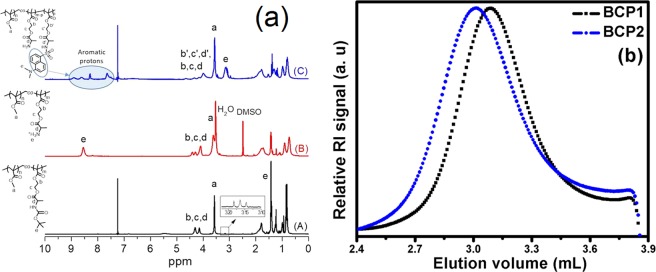
(**a**) The ^1^H NMR spectra of (**A**) P(MMA-*co*-Boc-Ala-HEMA) in CDCl_3_ (**B**) P(MMA-*co*-NH_3_^+^-Ala-HEMA) in DMSO-*d*_*6*_, and (**C**) dansyl tagged copolymer (**DCP2**) in CDCl_3_. (**b**) The GPC RI traces of **BCP1** and **BCP2**.

The CDP chain transfer agent produced polymers with controlled molecular weights and narrow *Ð* during the RAFT polymerizations of Boc-Ala-HEMA in DMF at 70 °C^31^. Thus, copolymerization reactions of MMA with Boc-Ala-HEMA were carried out *via* RAFT method by using CDP as the RAFT agent at [monomer (M)]/[CDP]/[AIBN] = 100:1:0.2. The synthesis scheme of this polymer is shown in Fig. 1. Polymers were characterized by GPC and NMR spectroscopy and results are summarized in Table 1. The GPC refractive index (RI) traces for both the copolymers are symmetric and unimodel (Fig. 2b). From the GPC study, number average molecular weight (*M*_n,GPC_) and *Ð* values were obtained for the copolymers and results are shown Table 1, which shows that theoretical molecular weights (*M*_n,theo_ = (([Monomer]/[CDP] × average molecular weight (MW) of monomer × Conv.) + (MW of CDP)) predicted from stoichiometry and monomer conversion matches well with the *M*_n,GPC_ values. Also, Table 1 shows narrow *Ð* values for both the copolymers.

**Table 1:** Experimental results from the RAFT copolymerization of MMA and Boc-Ala-HEMA in DMF at 70 °C for 8 h.

Polymer^a^	Boc-Ala-HEMA content in feed	Conv.^b^ (%)	Boc-Ala-HEMA content in copolymer^c^	*M*_n,theo_^d^ (g/mol)	*M*_n,GPC_^e^ (g/mol)	*Ð* ^e^	*M*_n,NMR_^f^ (g/mol)
**BCP1**	15	91	14	12100	11200	1.29	15000
**BCP2**	30	95	31	15600	11600	1.24	18400

^a^BCP means Boc protected copolymer. ^b^Determined by gravimetric analysis on the basis of the amount of monomer feed. ^c^Determined from ^1^H NMR analysis. ^d^*M*_*n*,*theo*_ = (([Monomer]/[CDP] × average molecular weight (MW) of monomer × conversion) + (MW of CDP)). ^e^Measured by GPC in THF. ^f^Calculated by ^1^H NMR from the integration ratio of the repeating unit protons to that of the polymer chain end protons.

The compositions of copolymers were determined from their ^1^H spectra recorded in CDCl_3_. All the peaks are assigned in Fig. 2a. The compositions of copolymers (Table 1) were determined from the integration ratio of intensities of the signals of the protons of Boc-Ala-HEMA units at 3.8–4.5 ppm (for –O–*CH*_*2*_–CH_2_–O– and chiral proton of alanine) to the –OC*H*_3_ protons at 3.59 ppm from MMA units. Comparison of the integration areas from the terminal –C*H*_*2*_– protons (from the –CH_2_–SC(=S)S–*CH*_*2*_–CH_2_– chain end) at 3.1–3.2 ppm and the repeating unit protons at 3.8–4.5 ppm (for –O–*CH*_*2*_–CH_2_–O– and chiral proton of alanine) allowed calculation of *DP*_n_ of Boc-Ala- HEMA unit in the copolymers (Fig. 2a(A)). Similarly, we have determined the *DP*_n_ of MMA unit in the copolymers from the ratio of integration areas at 3.l–3.2 ppm and at 3.59 ppm from –OC*H*_*3*_ unit in MMA. Number average molecular weight (*M*_n,NMR_) values of the P(MMA-*co*-Boc-Ala-HEMA) copolymers (Table 1) were determined from NMR analysis by using the formula: *M*_n,NMR_ = [(*DP*_*n*,MMA_ × *M*_MMA_) + (*DP*_*n*,Boc-Ala-HEMA_ × *M*_Boc-Ala-HEMA_) + molecular weight of CDP], where *DP*_n_ and *M* are the number average degree of polymerization and molecular weight of monomer, respectively^32^. Good agreement between the *M*_n,theo_ *M*_n,GPC_ and *M*_n,NMR_ molecular weights suggests RAFT polymerization (Table 1) occurs in controlled fashion. These polymers are soluble in most of the organic solvents but insoluble in water.

**Figure 3:**
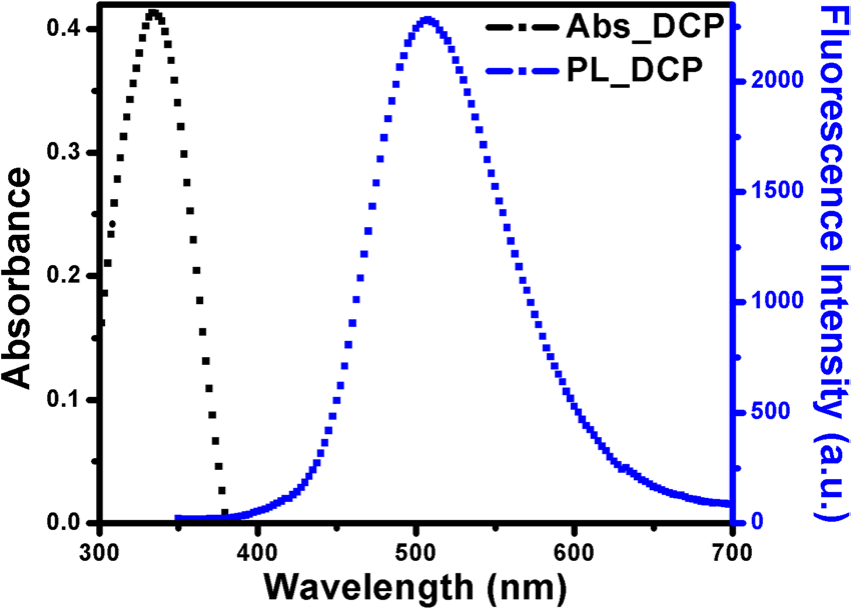
Absorption and emission spectra of dansyl tagged copolymer (**DCP**).

Next, Boc groups from the side chains of BCP1 and BCP2 were deprotected by reacting them with TFA in DCM at room temperature. Successful deprotection of Boc groups was confirmed from the disappearance of Boc-proton signal at about 1.42 ppm in the ^1^H NMR spectrum (Fig. 2a(B)). Then, P(MMA-*co*-NH_3_^+^-Ala-HEMA) was tagged with dansyl moiety. The ^1^H NMR spectrum of the dansyl tagged polymer P(MMA-*co*-Dansyl-Ala-HEMA) (**DCP**) CDCl_3_ indicates that aromatic protons associated with the dansyl moiety were appeared between 7.4–8.9 ppm (Fig. 2a(C)). The percentage of dansyl group incorporation into the **DCP1** and **DCP2** was determined by comparing the integration ratio of the peaks at *δ* = 4.00–4.45 ppm (5H, oxyethylene protons and chiral proton of alanine moiety) with those at 7.4–8.9 ppm (6H, aromatic protons) (Fig. 2a(C)). The percentage of dansyl group incorporation into the **DCP1** and **DCP2** were 72% and 81% respectively with respect to amino acid content into the copolymer. Note that dansyl chloride is non-fluorescent but when it reacts with primary or secondary amines it become fluorescent^33^. Therefore, the resulting dansyl tagged copolymer (**DCP1** and **DCP2**) are expected to be fluorescent. Here, we have used **DCP2** for our sensing experiments and hence forward will be called as **DCP**. The excitation and emission spectra of resulting dansyl tagged copolymer **DCP** was examined in THF at room temperature (Fig. 3), where absorption maxima (λ_abs_) at 334 nm and fluorescence emission maxima (λ_emiss_) at 505 nm are observed, which are characteristic of the green-yellow dansyl tag as described elsewhere^34^.

### Sensing analytes in solution

**Figure 4:**
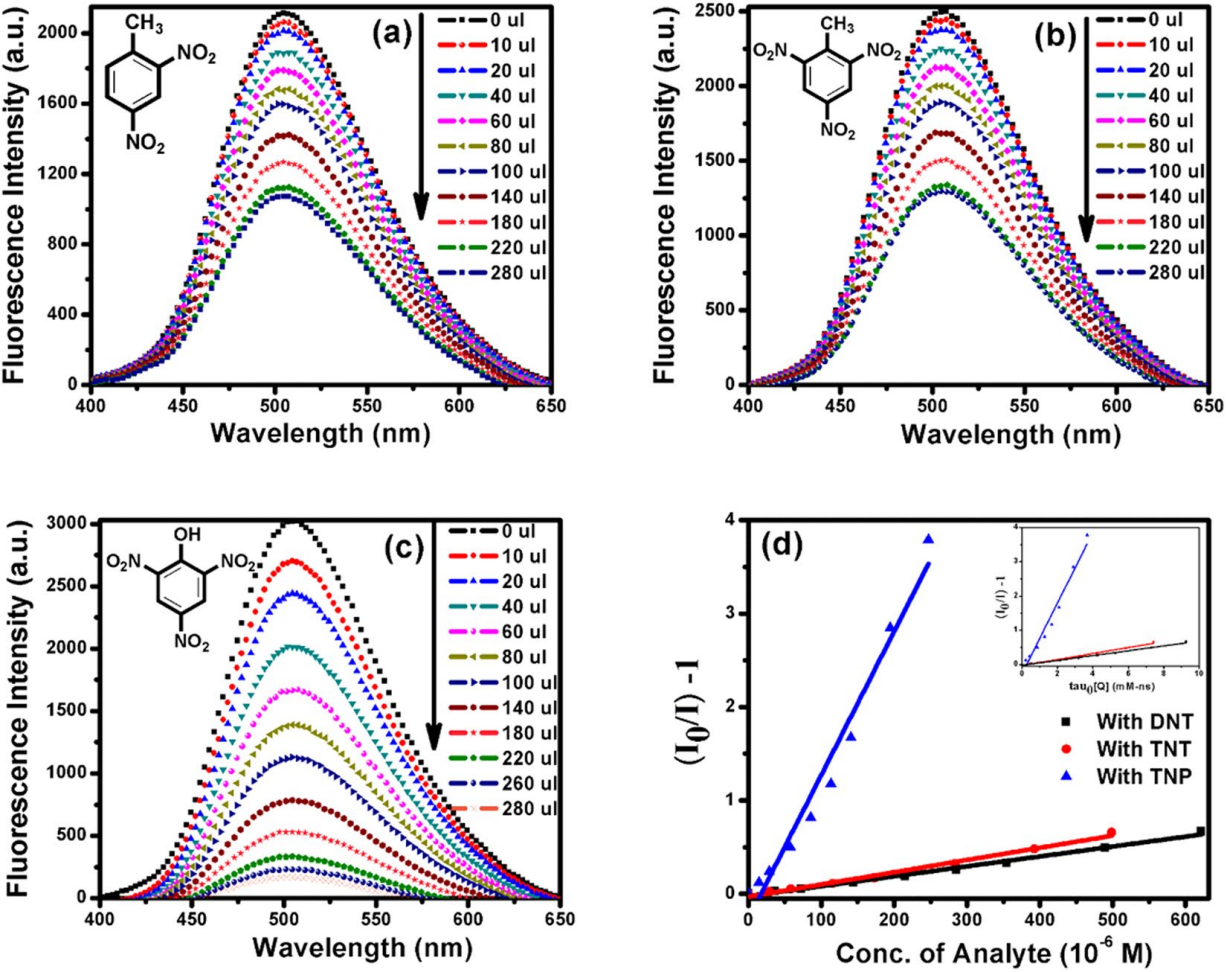
Fluorescence Quenching of **DCP** with addition of (**a**) DNT (**b**) TNT (**c**) TNP and (**d**) Stern-Volmer plot (I_0_/I − 1) v/s quencher conc.

The novel synthesized dansyl tagged copolymer (**DCP**) was used as optical sensors for sensing different NACs (such as DNT, TNT and TNP) and the detection method involved the measurement of the fluorescence signal of copolymer upon addition of different concentrations of analytes. Since our polymer **DCP** has limited solubility in water and **DCP** along with our studied analytes are well soluble in THF therefore we have chosen THF solvent to study the system. Figure 3 shows the absorption and the emission spectra of the dansyl tagged copolymer **DCP** respectively in THF solution. When excited at 334 nm, the synthesized **DCP** copolymer shows fluorescence emission from 400 nm to 650 nm with maximum around 505 nm. The absolute quantum yield (Φ_f_) of the co-polymer was measured to be 77% *via* the integrating sphere using an Edinburgh FLS980 fluorescence spectrometer. The fluorescence intensity of the co-polymer reduced drastically as shown in Fig. 4a–c respectively with the successive addition of NACs over a wide concentration range i.e. for DNT (36–937 μM), TNT (29–752 μM) and TNP (14–373 μM). The rapid reduction of fluorescence intensity with the increasing concentration of DNT, TNT and TNP suggests photo-induced energy transfer (PET) from the electron-rich dansyl groups on **DCP** copolymer chain to electron-deficient NAC molecules. The plausible mechanism for fluorescence quenching in presence of Nitroaromatics has been described in Fig. S1.

**Figure 5:**
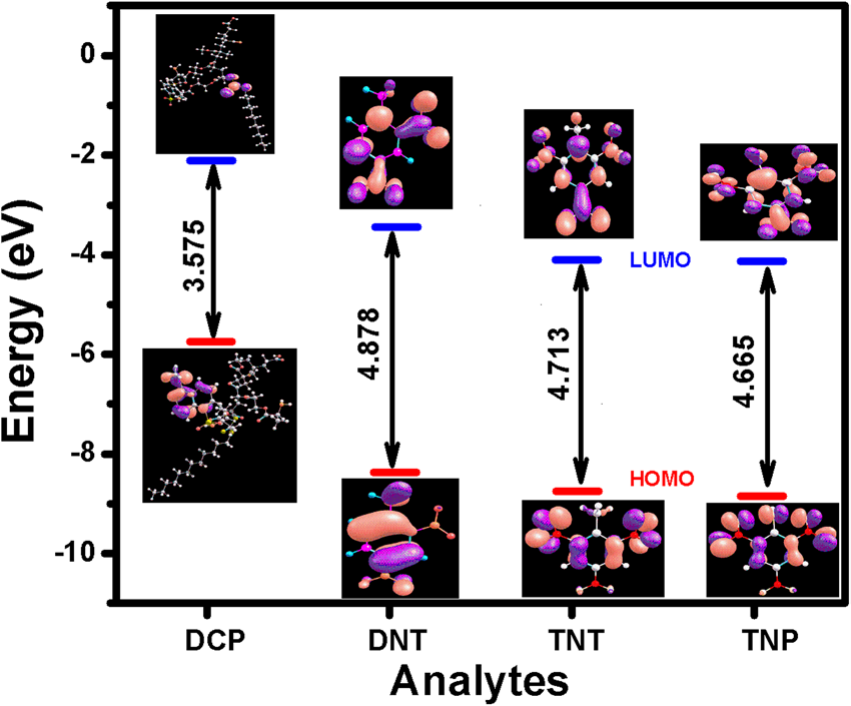
Calculated HOMO (red) and LUMO (blue) energy level diagram for **DCP** and NACs by DFT, frontier orbital theory using B3LYP functional and polarized 6–31 G⁄ basis set.

The sensitivity of fluorescence quenching is quantified by plotting Stern–Volmer plot given by equation (1).

1 $$\begin{array}{l}{{\rm{I}}}_{0}/{\rm{I}}=1+{{\rm{K}}}_{{\rm{SV}}}[{\rm{Q}}]\\ {{\rm{I}}}_{0}/{\rm{I}}=1+{{\rm{k}}}_{{\rm{q}}}{{\rm{\tau }}}_{0}[{\rm{Q}}]\end{array}$$

where, I and I_0_ are the fluorescence intensities of fluorophore with and without quencher molecules, [Q] is the quencher concentration, τ_0_ is the natural lifetime of fluorophore and K_SV_ & k_q_ are the proportionality constants known as Stern–Volmer constant & bimolecular quenching constant, which reflect the quenching efficiency or ease of access of the fluorophore to quencher^35,36^. A larger value of K_SV_ represents higher sensitivity of the fluorophore towards that particular analyte. Figure 4d shows the Stern–Volmer plot for DNT, TNT and TNP. The inset in Fig. 4d gives the value of bimolecular quenching constant (k_q_) (Fig. S2). The K_SV_ value was calculated to be 1.1 × 10^3^ M^−1^ and 1.3 × 10^3^ M^−1^ for DNT and TNT and a large value of 1.6 × 10^4^ M^−1^ for TNP. The fluorescence quenching efficiencies of these analytes follow the order TNP > TNT > DNT. The higher K_sv_ value for TNP compared to other NACs could be attributed to the higher energies of LUMO of **DCP** than NACs, (Fig. 5) and thus maintained a driving force for electron transfer from co-polymer (**DCP**) to electron deficient NACs, resulting in fluorescence quenching as calculated by Density Functional Theory (DFT) studies using B3LYP functional and polarized 6–31 G⁄ basis set (Table S1). The Table S1 have shown that LUMO energies were in good agreement with the maximum quenching observed for NACs (Figs 5 and S3), but the order of observed quenching efficiency is not fully in accordance with the LUMO energies of other NACs. This indicates that the photo-induced electron transfer (PET) is not the only mechanism for quenching.

On other hand, as per our observations, (I_0_/I) − 1 vs. [Q] plot deviates from linearity for high concentrations of NACs (Fig S4(a and b)). This deviation from linearity is an indication of the existence of two different quenching mechanism *viz*. static and dynamic quenching. In static quenching, there is a formation of a non-emissive complex between the analyte and fluorophore in the ground state itself. Whereas, in dynamic quenching, there is electron transfer between the analyte and the fluorophore in the excited state. In this case, the steady state Stern–Volmer equation is given by

2 $${{\rm{I}}}_{{\rm{0}}}/{\rm{I}}=(1+{{\rm{K}}}_{{\rm{C}}}[{\rm{Q}}])\,({\rm{1}}+{{\rm{K}}}_{{\rm{S}}}[{\rm{Q}}])$$

containing both collisional (K_c_) and static (K_s_) terms. The above equation can be rewritten as

3 $${{\rm{I}}}_{0}/{\rm{I}}=1+{{\rm{K}}}_{{\rm{C}}}[{\rm{Q}}]+{{\rm{K}}}_{{\rm{S}}}[{\rm{Q}}])+{{\rm{K}}}_{{\rm{C}}}{{\rm{K}}}_{{\rm{S}}}{[{\rm{Q}}]}^{2}$$

At low analyte concentration, the term [Q]^2^ is very less prominent and hence equation (3) will give a straight line. On the other hand, at higher concentration, the plot deviates from linearity and the effect of the collisional constant K_C_ will be significant^37^. The Stern–Volmer constant (Ksv) is the combination of Ks and Kc and can be obtained by linear fit for low concentration.

**Figure 6:**
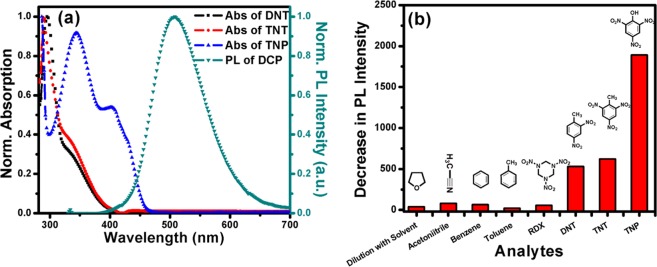
(**a**) Spectral overlap of the Norm. emission spectra of **DCP** with Norm. absorption spectra of different nitroaromatics. (**b**) Histogram for selectivity test of **DCP** with same concentration different analytes.

At the same time, in order to understand the other plausible mechanism behind this good sensing ability of the sensory system, the acidity order of the studied NACs (TNP > TNT > DNT) can be taken into consideration. Since TNP is a strong acid with a pKa value of ~0.38, it can easily dissociate *via* deprotonation of the strongly acidic phenolic –OH group in polar solvent (THF). This phenomenon in turn can accelerate the electrostatic attractive interactions between cationic **DCP** and anionic TNP because free amine group attached with the dansyl functionality get protonated in presence of anionic TNP. Consequently, the strong electrostatic interaction between cationic amine group and anionic TNP brings **DCP** and TNP in close proximity *via* electrostatic attraction and facilitates an efficient charge transfer and/or energy transfer mechanism, thereby providing an excellent fluorescence quenching with TNP, which will not happen in the cases of DNT and TNT. On other hand the high asymmetry of non-linear nature of Stern–Volmer plot for TNP (Fig. S4(b)) suggests Resonance Energy Transfer (RET) from the fluorophore **DCP** (donor) to non-emissive analytes TNP (acceptor) because the emission spectra of the **DCP** (donor) overlaps significantly with the absorption spectra of TNP (acceptor) and shown in Fig. 6a rendering the possibility of Resonance Energy Transfer (RET) from **DCP** (donor) to non-emissive TNP (acceptor) analyte. RET has moderately higher efficiency compared to the photo-induced electron transfer (PET) process as reported earlier^38^. To identify the extent of RET; overlap integral values for TNP, TNT and DNT were calculated. Table S2 confirms the highest integral value J(λ) (2.3 × 10^13^ M^−1^ cm^−1^ nm^4^) observed between the absorption spectrum of TNP and the emission spectrum of **DCP**, resulting in dramatic florescence quenching unlike other analytes (DNT and TNT) that have almost no overlap. The Förster distance R_0_ value obtained for **DCP**–TNP interaction was 25.3 Å. (Table S3) Therefore, **DCP** can detect TNP more selectively than TNT and DNT in solution. On further note, the estimated limit of detection (LOD) values were found to be 10.1, 9.1 and 3.7 µM for DNT, TNT and TNP, respectively (Table S4). The observed values are comparable to previous reported values (Table S5)^39–42^.

Since, the selectivity of a sensor is important especially in environmental samples to screen a large number of analytes, therefore we performed the fluorescence quenching experiment of the co-polymer with different other analytes such as acetonitrile, benzene, toluene and RDX. However, as shown in Fig. 6b, no appreciable change was observed. The concentration was adjusted to DNT concentration in each case. The solvent effect was eliminated with dilution test by adding similar amount of THF to the sensor system and no substantial decrease in fluorescence intensity was observed (Fig. 6b).

### Sensing mechanism

**Figure 7:**
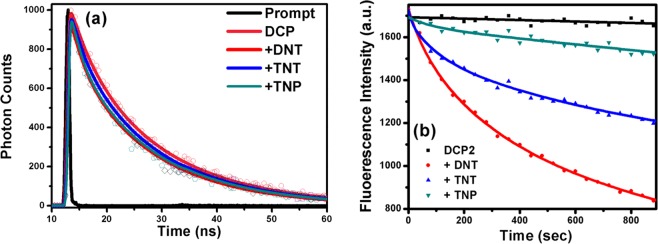
(**a**) Time resolved spectroscopic study of **DCP** with different nitroaromatics. (**b**) Quenching of fluorescence intensity of **DCP** thin film upon exposure to DNT, TNT and TNP saturated vapor.

**Figure 8:**
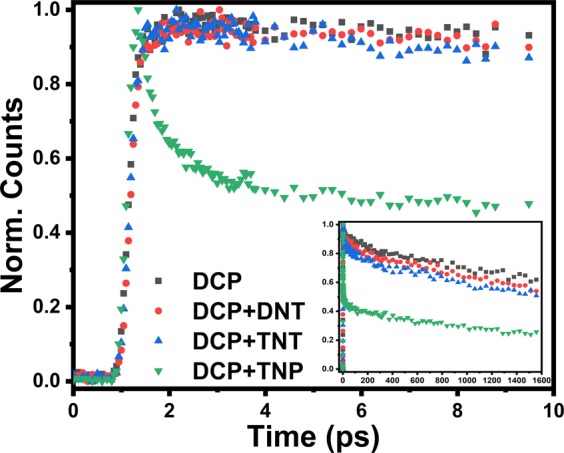
Femtosecond Transient Study of **DCP** (λ_Ex_ = 375 nm, λ_Em_ = 505 nm) with and without NACs (inset show the up-conversion signals collected in 1600 ps time window).

The fluorescence quenching requires molecular contact between the fluorophore and the quencher. This contact can be resulted from complex formation, which is static quenching or resulted from diffusive encounter, which is dynamic quenching^43^. The aforementioned two quenching processes can be distinguished by time-resolved measurements of the fluorescence decays of the fluorophore with or without quencher. For static quenching, formation of a non-fluorescent fluorophore–quencher complex is the origin of the fluorescence quenching. Here, any excited fluorophore molecule does not bound to the analyte and it will decay with their native natural lifetime. So, the lifetime of fluorophore will remain unchanged as the concentration of the quencher is increased^44^. For dynamic quenching (collisional quenching), collision of the quencher molecules to the excited fluorophores is a necessity, and thereby dynamic quenching is a diffusion controlled process and quenching occurs when a photoexcited fluorophore molecule interacts briefly with a colliding quencher molecule. Thus it results in a decrease in the average fluorescence lifetime of fluorophore molecule. The measurement of fluorescence lifetime change in the absence and in the presence of explosive quenchers represents the most prevalent way to examine whether the quenching is a static or dynamic process. Here, to confirm the interactive energy transfer between fluorophore and analytes, Time resolved spectroscopy was performed to measure the fluorescence lifetime by monitoring emission of fluorophore at 505 nm in the absence and in the presence of analytes (Fig. 7a). The fluorescence data of **DCP** copolymer is reliably fitted to a double exponential decay function. The decay processes consist of a fast component of 3.86 ns (19%) and a slow component of 15.5 ns (81%) with an average lifetime of 14.9 ns. Upon addition of DNT, TNT and TNP the average lifetime of **DCP** copolymer reduce to 13.9 ns, 13.9 ns and 13.8 ns respectively. (Table 2) This indicates a dynamic quenching process between **DCP** and DNT, TNT and TNP but the only 7% drop in average lifetime of DCP copolymer in presence of NACs suggest that here fluorescence quenching can be caused by the combination of collisional and static quenching, and not by only dynamic quenching. This is also indicated by non-linearity in Stern–Volmer plot for DNT, TNT and TNP (Fig. S4(a and b)). Signal of ground state complex formation in UV-Vis Spectra of fluorophore i.e. due to perturbation by analyte reflect the possibility of static quenching. For our observation, we didn’t find any such signal in UV-Vis Spectra (Fig. S5), however, it doesn’t exclude the possibility of static quenching. On the other hand, the photo induced electron transfer is a nonradiative decay process which occurs in much faster time scale and beyond the detection limit of TCSPC (200 ps). So, to resolve the ultrafast component, we did ultrafast fluorescence spectroscopy of **DCP** *via* up-conversion in the absence and presence of DNT, TNT and TNP. Figure 8 shows the fluorescence up-conversion signals of **DCP** without and with the NACs in femtoseconds (fs) time resolutions. The up-conversion signals were reliably fitted with a quad-exponential decay function in all cases (Table 3, Fig. S6(a–d)). The decay process of copolymer **DCP** consists of two fast components with time constants 0.3 and 11.88 ps having relative amplitudes a1 = −0.19 and a2 = 0.07, respectively. Along with the fast components, the decay process also consists of two slow components with 603.08 and 6254.6 ps having relative amplitudes a3 = 0.12 and a4 = 0.77, respectively. Upon addition of DNT, TNT and TNP there is a major decrease in slowest component from 6254.6 ps to 4351.3, 3727.2 and 2717.5 ps, respectively. It is also seen from the fluorescence upconversion experiment (Fig. 8) that the static quenching is predominant for the lower concentrations of NACs and mixed quenching (static and dynamic) is present at higher concentration. Along with this, a rapid decay in a1 component of DCP + TNP provide a robust agreement to the possibility of Resonance Energy Transfer (RET) from **DCP** to TNP as indicated in Fig. 8.

**Table 2:** Fluorescence decay lifetime data of **DCP** with and without NACs.

Components	A1	A2	T1 (ns)	T2 (ns)	Avg. Lifetime (ns)
DCP	0.19	0.81	3.86	15.50	14.9
DCP + DNT	0.24	0.76	2.61	14.50	13.9
DCP + TNT	0.19	0.81	2.46	14.41	13.9
DCP + TNP	0.26	0.74	1.97	14.36	13.8

**Table 3:** Femtosecond Decay Parameters of **DCP** emission (at 505 nm) with and without NACs.

Components	a1^#^	t1 (ps)	a2	t2 (ps)	a3	t3 (ps)	a4	t4 (ps)
DCP	−0.19	0.30	0.07	11.88	0.12	603.08	0.77	6254.6
DCP + DNT	−0.21	0.23	0.11	23.69	0.32	2658.3	0.51	4351.3
DCP + TNT	−0.96	0.10	0.11	7.07	0.09	105.51	0.76	3727.2
DCP + TNP	0.67	0.32	0.19	2.13	0.06	57.37	0.41	2717.5

^#^A negative amplitude value indicates the corresponding component is rise rather than decay.

### Thin film sensing

The suitability of our fluorescent sensor to nitroaromatics vapor was also tested. Figure S7(a and b) depict the absorption and emission spectra of **DCP** respectively in both solution and thin film. The permeation of the analyte in the **DCP** film is the most important parameter in determining the fluorescence quenching response time and consequently the time dependence of the fluorescence intensity from **DCP** film for a series of nitroaromatics was studied. As shown in Fig. 7b the initial fluorescence intensity of the 20 nm thin film of **DCP** was quenched to 19% for DNT, 13% for TNT and 4% for TNP in just 2 min. The responsively of sensing is in the order DNT > TNT > TNP. The higher fluorescence quenching by DNT compared to TNT and TNP could be attributed to the higher vapor pressure of DNT than TNT and TNP at room temperature (25 °C) even though their sensitivity in solution are different^45^. Previously, Yang and Swager in their study of pentiptycene-containing poly(phenyleneethynylene)s and Yao Liu and Kirk S. Schanze in poly[1-phenyl-2-(4-trimethylsilylphenyl)ethyne] thin film sensing study observed the similar quenching behavior in vapor phase^46,47^.

To demonstrate a practical application of **DCP**, A paper sensor was designed and tested for the saturated vapor of DNT. In a control experiment with filter paper under 365 nm UV illumination, the fluorescence intensity of **DCP** was quenched drastically in the environment of saturated vapor of DNT with immediate effect (Fig. S8). It could be observed clearly with even naked eyes and this study led us to conclude that the **DCP** paper sensor would be a potential sensor for the instant visualization and onsite detection of traces of NACs by a very simple practical method.

## Experimental Section

### Materials and methods

Dicyclohexylcarbodiimide (DCC, 99%), 4 dimethylaminopyridine (DMAP, 99%), 2-hydroxyethyl methacrylate (HEMA, 97%), N,N-dimethylformamide (DMF, 99.9%) and dansyl chloride (98%) were purchased from Sigma and used without any further purification. Boc-L-alanine (Boc-L-Ala-OH, 99%) and trifluoroacetic acid (TFA, 99.5%) were purchased from Sisco Research Laboratories Pvt. Ltd., India and used as received. Methyl methacrylate (MMA) (Sigma, 99%) was passed through a basic alumina column prior to polymerization 2,2′-Azobisisobutyronitrile (AIBN, Sigma, 98%) was recrystallized from methanol. 4-Cyano-(dodecylsulfanylthiocarbonyl)sulfanylpentanoic acid (CDP) was synthesized following the standard literature procedure^31^. The alanine based vinyl monomer, Boc-L-Alanine methacryloyloxyethyl ester (Boc-Ala-HEMA), was synthesized by the coupling reaction of Boc-L-Ala-OH with HEMA in the presence of DCC and DMAP as reported previously^48^. NMR solvents such as CDCl_3_ (99.8% D) and dimethylsulfoxide-d_6_ (DMSO-d_6_) (99.8% D) were obtained from Cambridge Isotope Laboratories, Inc., USA. The solvents dichloromethane (DCM), methanol (MeOH), ethyl acetate (EtOAc), chloroform and hexanes (mixture of isomers) were purified by following general procedure.

Molecular weights and molecular weight distributions (dispersity (*Đ*)) of polymers were determined by waters ACQUITY Advanced Polymer Chromatography (APC). The instrument contains a 1500 series HPLC pump, an ACQUITY® refractive index (RI) detector, one ACOUITY APC^TM^ XT 2002.5 μm (4.6 × 7.5 mm) column in THF at 45 °C at 0.25 mL/min flow rate. Poly(methyl methacrylate) (PMMA) standards were used to calibrate the instrument. ^1^H NMR spectra were acquired in a Jeol spectrometer operating at 400 MHz.

### Synthesis of copolymer

All polymerization reactions were carried out in the presence of AIBN as initiator and DMF as solvent at 70 °C. A representative example is as follows: MMA (522.0 mg, 5.21 mmol), Boc-Ala-HEMA (278.0 mg, 0.92 mmol), AIBN (2.0 mg, 0.01 mmol), CDP (24.0 mg, 0.06 mmol) and DMF (2.4 g) were placed in a 20 mL reaction vial equipped with a magnetic stir bar. The vial was purged with dry N_2_ for 15 min and placed in a preheated reaction block at 70 °C. The feed ratios of MMA and Boc-Ala-HEMA were varied to get copolymers of various compositions and we named those polymers as **BCP1** and **BCP2** (Table 1). After 8 h, the polymerization reaction was stopped by cooling the vial in an ice-water bath and exposed to air. Finally, the copolymer was purified by repeated precipitation in hexanes (non-solvent) from the acetone solution to get yellowish solid polymer.

### Deprotection of Boc groups

In the next stage, deprotection of Boc groups was carried out by adding 1.5 mL TFA into a 20 mL glass vial containing 0.5 g of copolymer (**BCP**) in 1.5 mL DCM at room temperature (Fig. 2). The solution was stirred for 2 h to obtain free primary amine containing polymer, P(MMA-*co*-NH_3_^+^-Ala-HEMA). The product was purified by repeated precipitation in diethyl ether/acetone solution and dried under high vacuum at 45 °C for 10 h.

### Dansyl group labelling

In a typical example, 400 mg (0.69 mmol with respect to NH_3_^+^-Ala-HEMA) of P(MMA-*co*-NH_3_^+^-Ala-HEMA) was dissolved in 2 mL dry DCM in a 20 mL glass vial. Triethylamine (104.0 mg, 1.03 mmol) and dansyl chloride (279.0 mg, 1.03 mmol) were then added to the vial in an ice-water bath under stirring and was allowed to react at room temperature for 12 h. The polymer solution was concentrated using rotary evaporator and then precipitated into hexanes for several time and dried under high vacuum at 45 °C for 10 h. Deprotection of Boc groups and subsequent dansyl labelling from **BCP1** and **BCP2** gave **DCP1** and **DCP2**, respectively.

### Fluorescence quenching and lifetime study

The detection of NACs, based on fluorescence quenching by photo-induced electron-transfer (PET) mechanism, was studied with the dansyl tagged copolymer P(MMA-*co*-Dansyl-Ala-HEMA) (**DCP**). Sensing study was performed with **DCP2**, since the **DCP2** is more fluorescent and have single isolated prominent emission spectrum in the visible region compare to **DCP1**, though we have chosen **DCP2** as our sensor material and abbreviated as **DCP** for further investigations, started with UV-Vis absorption spectra (using Lasany LI-2800 UV-Vis double beam spectrophotometer) and steady state fluorescence emission spectra (using Shimadzu RF-6000 Spectro Fluorometer). The fluorescence quenching was initially investigated in solution of **DCP**, THF as solvent, with the successive addition of DNT, TNT and TNP. We have also investigated the effect of saturated vapor of DNT, TNT and TNP, for this DNT (23 mg), TNT (35 mg) and TNP (121 mg) was put in a quartz cuvette and sealed it for 36 hours so that the vapor can saturate. Then the thin film of **DCP** coated using drop cast method on microbiological slide was inserted in that sealed cuvette and the fluorescence data were recorded in real time with Spectro Fluorometer immediately after exposing the copolymer film to the vapor of analyte. Fluorescence lifetime measurement of **DCP** alone and with nitroaromatics analyte (DNT, TNT and TNP) was recorded by time-correlated single photon counting (TCSPC) (using Horiba Jobin Yvon, Florocube with excitation Sources: Nano LEDs with wavelength of 340 nm). The decays in fast time scales were measured using fluorescence up-conversion (UPC) setup (FOG100, CDP) pumped with a femtosecond Ti:sapphire laser (MaiTaiHP, Spectra-Physics). The time resolution (IRF) for the UPC setup was ~350 fs (measured with cross-correlation of Raman signal from ethanol). Selectivity test with different analyte and dilution test was also performed by adding equal amount in solution of **DCP**.

## Conclusion

In summary, we have synthesized a fluorescent dansyl tagged copolymer (Φ_f_ = 77%) by using controlled RAFT polymerization. The excitation and emission maximum of **DCP** copolymer were observed at 334 nm and 505 nm (334 nm excitation) in THF as solvent. In solution, fluorescence quenching was recorded with trace amount of DNT, TNT and TNP and Stern-Volmer constant (K_SV_) was calculated to be 1.1 × 10^3^ M^−1^, 1.3 × 10^3^ M^−1^ and 1.6 × 10^3^ M^−1^. This quenching phenomenon is the combination of static and dynamic which is confirmed by fluorescence up-conversion and time-resolved fluorescence data. In presence of DNT, TNT and TNP vapor, the fluorescence of the thin film of **DCP** is quenched by 19%, 13% and 4% respectively in just 2 min at room temperature.

The sensing capability of **DCP** for both solution and vapor, demonstrates its distinguished potential as an efficient fluorescent sensor for the detection of nitro-aromatic explosives for future field based study. Thin film of **DCP** integrated with other technologies such as electronics, imaging, and sensor design could play more important roles in real explosive detection such as buried landmines, and for monitoring environmental contamination in soil, groundwater and seawater.

## Supplementary information

Supplimentary Information
