# Supplementary material for: Multimodal Fluorescent Polymer Sensor for Highly Sensitive Detection of Nitroaromatics
Source: Sci Rep. 2019 May 13;9:7269. doi: 10.1038/s41598-019-43836-w (PMC6514181; doi:10.1038/s41598-019-43836-w)
Supplement: Supplementary file 1 — Supplimentary Information [file 41598_2019_43836_MOESM1_ESM.docx]

Supporting information for

Multimodal Fluorescent Polymer Sensor for Sensitive Detection of Nitroaromatics

Vishal Kumar^1^, Binoy Maiti^2^, Mrinmoy Kumar Chini^1^, Priyadarsi De^2^, Soumitra Satapathi^1,*^

^1^ Department of Physics, Indian Institute of Technology Roorkee, Roorkee, Uttarakhand, 247667, India.

^2^ Polymer Research Center, Department of Chemical Science, Indian Institute of Science Education and Research Kolkata Mohanpur, 741246.

*Corresponding Author: [ssphf.fph@iitr.ac.in](mailto:ssphf.fph@iitr.ac.in)


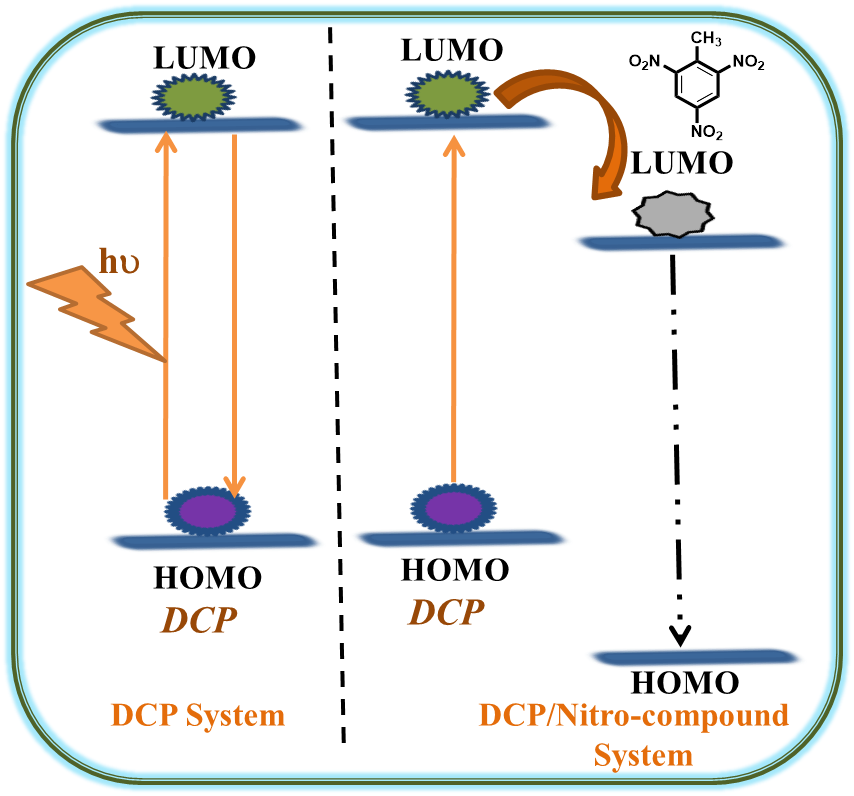


**Figure [S1]** Fluorescence quenching mechanisms of Fluorophore with Nitroaromatic analytes.

**

**

Figure [S2] Plot for non-radiative rate constant (k_q_) using Equ. I_0_/I = 1+k_q_τ_0_ [Q].


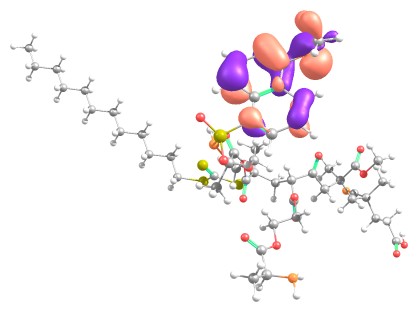

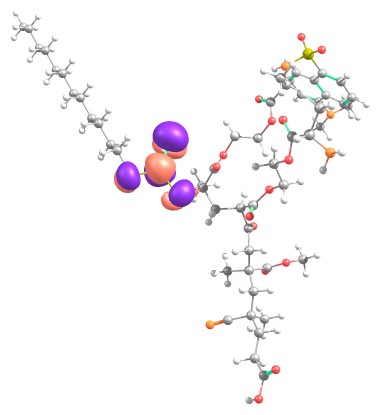


***LUMO***

***HOMO***

Figure [S3] HOMO and LUMO distribution of Energy Minimized Structures of three repeating unit of Densyl Group of Copolymer (DCP) obtained by the DFT calculation preliminary insight of the frontier orbital energy levels, using B3LYP functional and polarized 6–31G⁄ basis set.

**Table S1.** Homo and LUMO energies calculated for DCP and different analytes (Calculated by DFT, frontier orbital theory using B3LYP functional and polarized 6–31G⁄ basis set).

| Analytes | HOMO (eV) | LUMO (eV) | Band gap (eV) |
| --- | --- | --- | --- |
| DCP | -5.671 | -2.096 | 3.575 |
| 2,4-Dinitrotoluene (DNT) | -8.347 | -3.468 | 4.878 |
| 2,4,6-Trinitrotoluene (TNT) | -8.743 | -4.029 | 4.713 |
| 2,4,6-Trinitrophenol (TNP) | -8.808 | -4.143 | 4.665 |


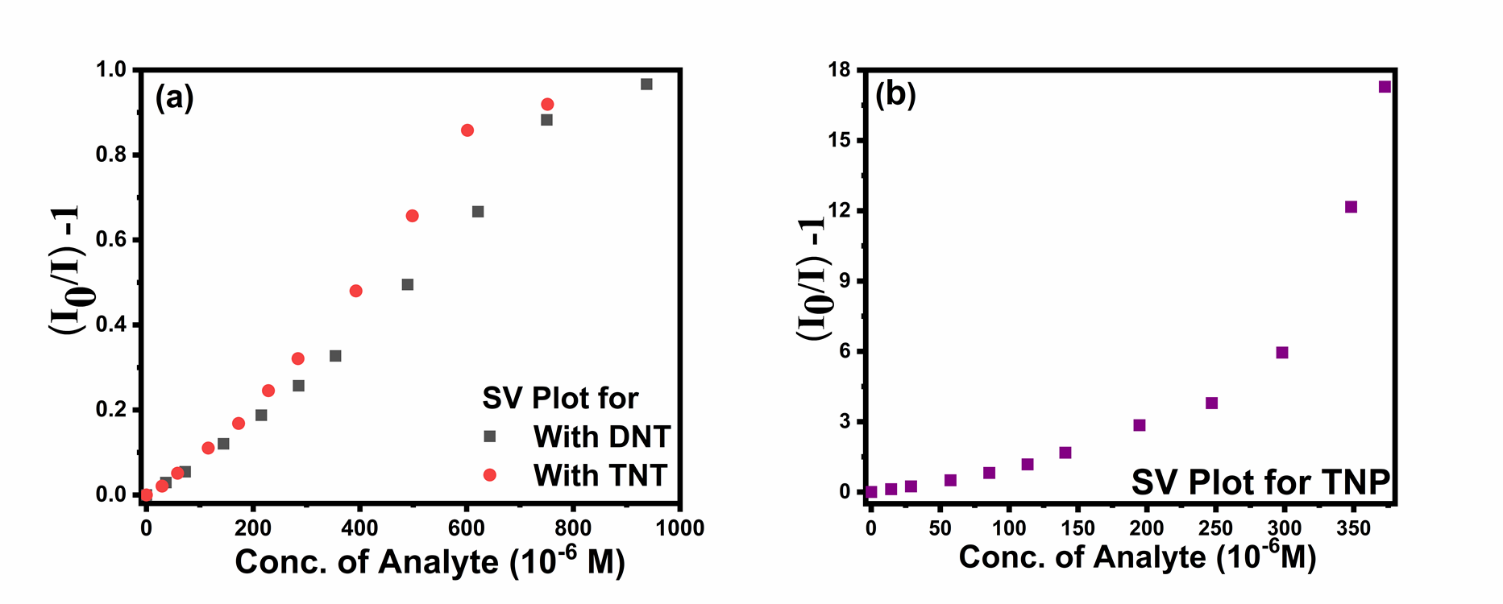


Figure [S4] Stern-Volmer plot (I_0_/I – 1) v/s quencher conc. for (a) DNT, TNT and (b) TNP.





Figure [S5] Absorption spectra of the DCP with or without nitroaromatic molecules.

# Calculation of Overlap Integral Values and Förster Radius

To identify the extent of energy transfer, overlap integral values for all analytes were calculated using the equation 1 shown below^1^

………………. (1)


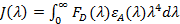

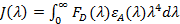


where, *F_D_ (λ)* denotes the corrected fluorescence intensity of donor in the range of *λ* to *λ + Δλ* with the total intensity normalized to unity, *Ɛ_A_* is molar absorptivity of the acceptor at *λ* in M^-1^ cm^-1^. The Förster distance *R_0_* was also calculated for dipole-dipole interaction of **DCP** with different analytes using the equation shown below

……..………… (2)


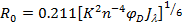

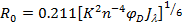


where, *J_λ_* is the degree of spectral overlap between donor fluorescence spectrum and the acceptor absorption spectrum, *ϕ_D_* is fluorescence quantum yield of the donor (without acceptor), *n* is the refractive index of the medium and *K^2^* is the dipole orientation factor for the emission and absorption and its value depends on their relative orientation, usually taken as 2/3. For our present system *n* =1.407, and *ϕ_D_* =0.77.

**Table S2.** Overlap integral *J (λ)* values obtained for various analytes.

| Analytes | *J (λ)* (M^-1^ cm^-1^ nm^4^) |
| --- | --- |
| 2,4-Dinitrotoluene (DNT) | 9.319 × 10^11^ |
| 2,4,6-Trinitrotoluene (TNT) | 2.294 × 10^12^ |
| 2,4,6-Trinitrophenol (TNP) | 2.289 × 10^13^ |

**Table S3.** Förster distance R_0_ values obtained for various analytes.

| Analytes | *R_0_* (Å) |
| --- | --- |
| 2,4-Dinitrotoluene (DNT) | 14.861 |
| 2,4,6-Trinitrotoluene (TNT) | 17.268 |
| 2,4,6-Trinitrophenol (TNP) | 25.337 |

# Calculation of Detection Limit

# The limit of detection (LOD) was estimated using the equation 3σ/K, where σ denotes the standard deviation of intensity response and K represents slope of calibration curve.

**Table S4.** Limit of detection (LOD) values obtained for various analytes.

| Analytes | Detection Limit |
| --- | --- |
| 2,4-Dinitrotoluene (DNT) | 10.1 µM (1.84 ppm) |
| 2,4,6-Trinitrotoluene (TNT) | 9.1 µM (2.07 ppm) |
| 2,4,6-Trinitrophenol (TNP) | 3.7 µM (0.85 ppm) |

**Table S5.** A comparative study of some conjugated polymers-based reports for Nitroaromatic explosive detection.

| \| **Publication** \|  \|  \|  \|  \| \| --- \| --- \| --- \| --- \| --- \| | **K_SV_ (M^-1^)** | **Detection Limit** | **Selectivity** | **Sensing Mechanism** |
| --- | --- | --- | --- | --- | --- | --- | --- | --- | --- |
| ***Present Manuscript*** | 1.1×10^3^ **(DNT)**  1.3×10^3^ **(TNT)**  1.6×10^4^**(TNP)** | 10.1 µM **(DNT)**  9.1 µM **(TNT)**  3.7 µM **(TNP)** | **Selective** | Electron Transfer |
| Nanoscale,2014,6, 2608-2612 | 2.36×10^3^ **(DNT)** | 0.341 mM **(DNT)** | NA | Electron transfer |
| Nanoscale, 2015,7, 1872-1878 | NA | 0.30 μM **(TNP)** | Selective | Electrostatic  interaction |
| Analyst 2018, 143, 1036-1041 | 3.1×10^3^ **(DNT)**  9.1×10^3^ **(TNT)** | 7.6 µM **(DNT)**  2.5 µM **(TNT)** | Selective | Aggregation-induced emission enhancement (AIEE) |
| Sensors and Actuators B 2018, 262, 298-305 | 1.42×10^5^**(TNP)** | 7.04×10^−7^mol/L **(TNP)** | Selective | Molecular interactions |


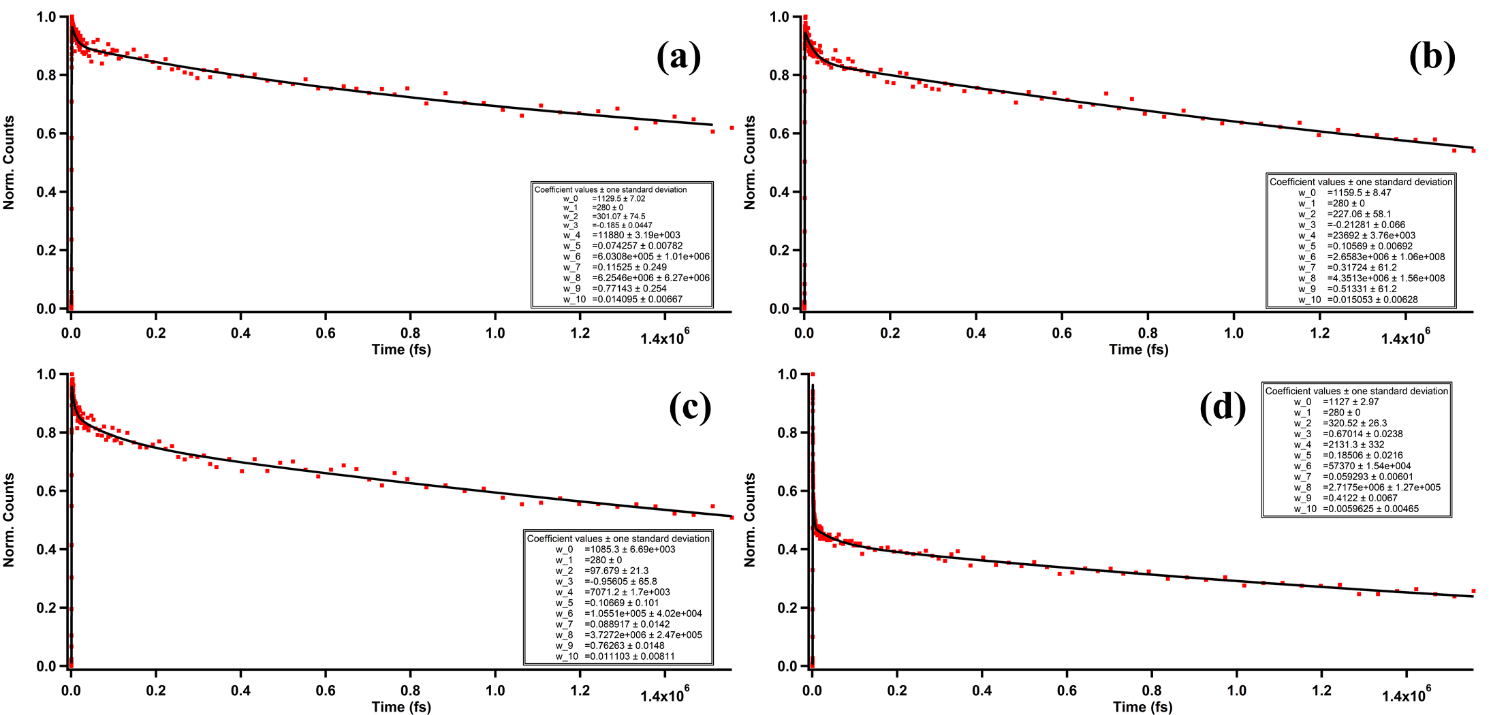


**Figure [S6]** Data fit for Femtosecond Transient Study of **(a)** only **DCP** and **DCP** in presence of **(b)** DNT, **(c)** TNT and **(d)** TNP (λ_Ex_=375 nm, λ_Em_=505 nm).





**Figure [S7] (a)** Absorption and **(b)** Emission spectra of DCP in THF solution and thin film.


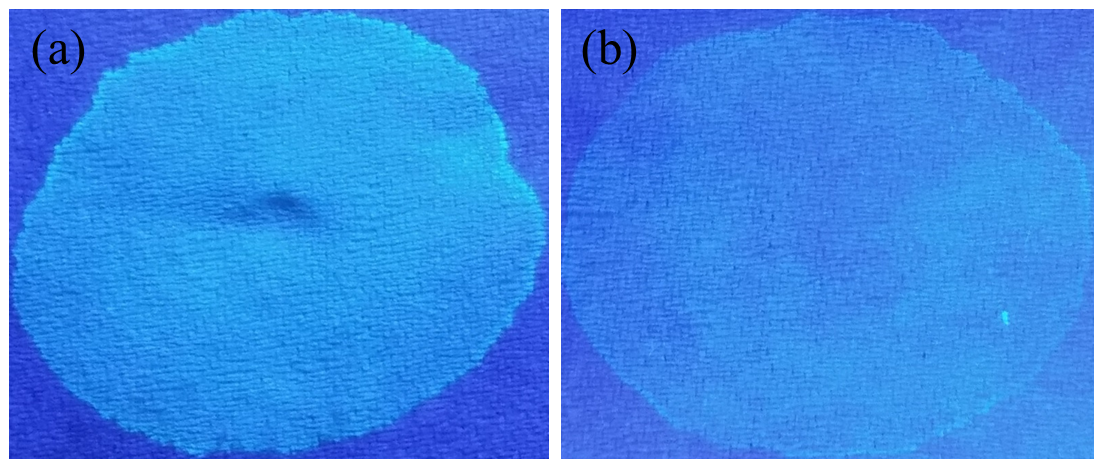


Figure [S8] Luminescent photographs of filter papers impregnated with DCP (a) before and (b) after exposure to the saturated vapor of DNT. Photographs were taken under 365 nm UV illumination.

REFERENCES

[1] Lakowicz, J. R. 2006, *Principles of Fluorescence Spectroscopy,* 3^rd^ Edition, Springer.

[2] Shrivastava, A. & Gupta, V.B. Methods for the determination of limit of detection and limit of quantitation of the analytical methods. *Chronicles of Young Scientists* **2**, 21-25, doi: 10.4103/2229-5186.79345 (2011).
